# *N*,*N*′-Dibutyl-*N*,*N*,*N*′,*N*′-tetra­methyl­ethane-1,2-diaminium μ-oxido-bis­[trichloridoferrate(III)]

**DOI:** 10.1107/S1600536812019964

**Published:** 2012-05-12

**Authors:** Sari M. Närhi, Jatta Kostamo, Janne Asikkala, Raija Oilunkaniemi, Risto S. Laitinen

**Affiliations:** a Department of Chemistry, PO Box 3000, FI-90014 University of Oulu, Finland

## Abstract

The asymmetric unit of the title compound, (C_14_H_34_N_2_)[Fe_2_Cl_6_O], contains one complete cation, two half-cations and two anions. The two half-cations are completed by crystallographic inversion symmetry. The Fe^III^ atoms adopt fairly regular FeCl_3_O tetra­hedral geometries, although the bridging Fe—O—Fe bond angles differ significantly in the two anions, which both adopt an eclipsed conformation. In the crystal, the components are linked by C—H⋯Cl and C—H⋯O inter­actions.

## Related literature

For the structure of the bromide salt of the same cation, see: Hattori *et al.* (1998 ▶).
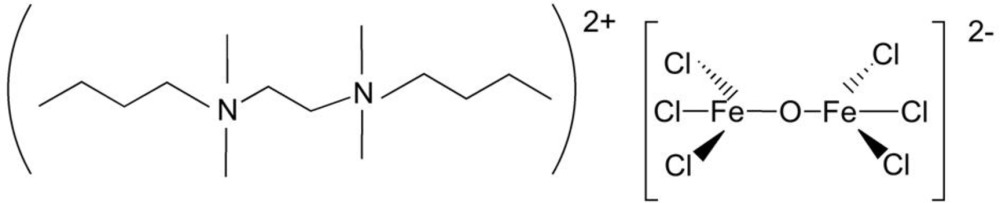


## Experimental

### 

#### Crystal data

(C_14_H_34_N_2_)[Fe_2_Cl_6_O]
*M* *_r_* = 570.83
Triclinic, 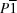

*a* = 8.9803 (18) Å
*b* = 14.689 (3) Å
*c* = 19.249 (4) Å
α = 81.75 (3)°
β = 87.66 (3)°
γ = 80.32 (3)°
*V* = 2476.8 (9) Å^3^
*Z* = 4
Mo *K*α radiation
μ = 1.83 mm^−1^
*T* = 120 K
0.25 × 0.10 × 0.08 mm

#### Data collection

Bruker–Nonius KappaCCD diffractometer
Absorption correction: multi-scan (*SHELXTL*; Sheldrick, 2008 ▶) *T*_min_ = 0.658, *T*_max_ = 0.868
29910 measured reflections
8498 independent reflections
7321 reflections with *I* > 2σ(*I*)
*R*_int_ = 0.096

#### Refinement

*R*[*F* ^2^ > 2σ(*F* ^2^)] = 0.045
*wR*(*F* ^2^) = 0.128
*S* = 1.12
8498 reflections
464 parameters
H-atom parameters constrained
Δρ_max_ = 0.85 e Å^−3^
Δρ_min_ = −0.81 e Å^−3^

### 

Data collection: *COLLECT* (Bruker, 2008 ▶); cell refinement: *DENZO-SMN* (Otwinowski & Minor, 1997 ▶); data reduction: *DENZO-SMN*; program(s) used to solve structure: *SHELXS97* (Sheldrick, 2008 ▶); program(s) used to refine structure: *SHELXL97* (Sheldrick, 2008 ▶); molecular graphics: *DIAMOND* (Brandenburg, 1999 ▶); software used to prepare material for publication: *WinGX* (Farrugia, 1999 ▶).

## Supplementary Material

Crystal structure: contains datablock(s) I, global. DOI: 10.1107/S1600536812019964/hb6765sup1.cif

Structure factors: contains datablock(s) I. DOI: 10.1107/S1600536812019964/hb6765Isup2.hkl

Additional supplementary materials:  crystallographic information; 3D view; checkCIF report

## Figures and Tables

**Table:** 

Fe1—O1	1.776 (3)
Fe1—Cl12	2.2060 (12)
Fe1—Cl13	2.2130 (12)
Fe1—Cl11	2.2344 (12)
Fe2—O1	1.773 (3)
Fe2—Cl21	2.2183 (12)
Fe2—Cl22	2.2321 (13)
Fe2—Cl23	2.2356 (13)
Fe3—O2	1.756 (3)
Fe3—Cl33	2.2141 (13)
Fe3—Cl31	2.2257 (12)
Fe3—Cl32	2.2316 (13)
Fe4—O2	1.765 (3)
Fe4—Cl42	2.2142 (12)
Fe4—Cl43	2.2248 (12)
Fe4—Cl41	2.2338 (13)

**Table:** 

Fe2—O1—Fe1	142.68 (16)
Fe3—O2—Fe4	160.84 (18)

**Table 2:** Hydrogen-bond geometry (Å, °)

*D*—H⋯*A*	*D*—H	H⋯*A*	*D*⋯*A*	*D*—H⋯*A*
C2—H2*A*⋯Cl13^i^	0.99	2.80	3.336 (3)	114
C2—H2*A*⋯Cl22^i^	0.99	2.76	3.656 (4)	150
C3—H3*B*⋯Cl42^ii^	0.98	2.82	3.688 (4)	148
C3—H3*C*⋯Cl13^i^	0.98	2.77	3.746 (4)	176
C9—H9*A*⋯Cl32^iii^	0.98	2.79	3.708 (4)	155
C15—H15*B*⋯Cl33^ii^	0.99	2.69	3.623 (4)	157
C16—H16*A*⋯Cl32	0.98	2.80	3.738 (5)	160
C17—H17*A*⋯Cl23^iv^	0.98	2.69	3.627 (4)	161
C22—H22*A*⋯O1^v^	0.99	2.39	3.336 (4)	161
C24—H24*C*⋯Cl11^iv^	0.98	2.78	3.677 (3)	152
C26—H26*A*⋯Cl13^vi^	0.99	2.76	3.713 (4)	162

Symmetry codes: (i) 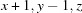; (ii) 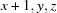; (iii) 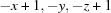; (iv) 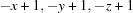; (v) 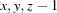; (vi) 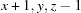.

**Fig. 1.**
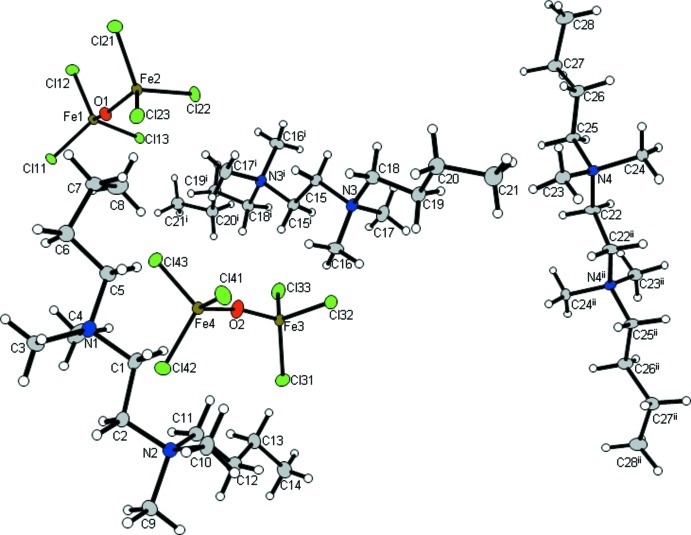
The molecular structure of the title compound with displacement ellipsoids drawn at 50% probability. Symmetry code: (i) -x + 1, -y + 1, -z + 1; (ii) 1 - x, 1 - y, -z.

**Fig. 2.**
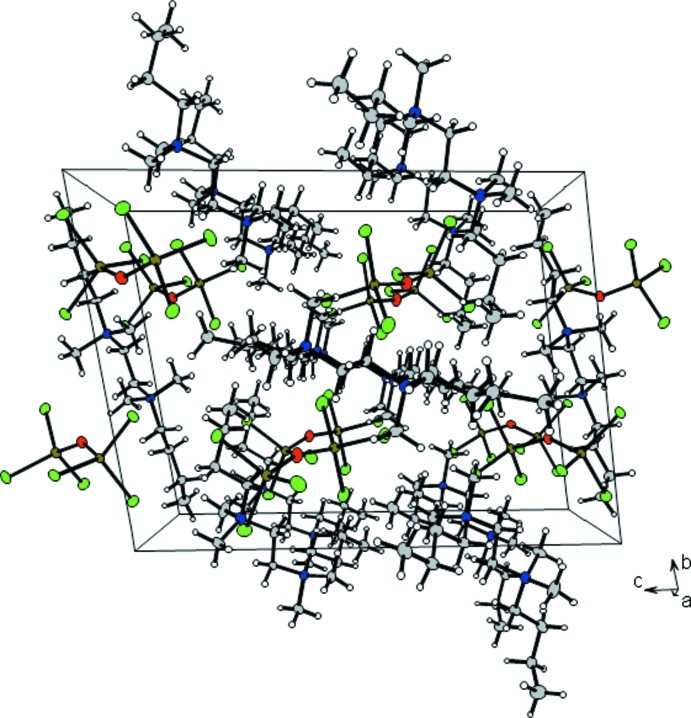
The packing of the molecules viewed along a axis.

## supplementary crystallographic information

### Comment

The asymmetric unit of (C_14_H_34_N_2_)[Fe_2_Cl_6_O] consists of one full cation and two halves of cations and two anions (see Fig. 1). The N—C bond lengths in the cations range from 1.492 (5) to 1.538 (5) Å and the C—C bond lengths from 1.511 (5) to 1.537 (5) Å. These can be compared to the bond lengths in the bromide salt of the corresponding cation (Hattori *et al.* 1998).

The Fe—Cl bond lengths in the anions range from 2.2060 (12) to 2.2356 (13) Å and the Fe—O bond lengths from 1.756 (3) to 1.776 (3) Å. The Fe1—O1—Fe2 and Fe3—O2—Fe4 bond angles are 142.68 (16) and 160.84 (18) °, respectively. All bond lengths and angles are quite normal. The closest internuclear contact from O1 is 2.387 (3) Å to H22A and from O2 2.752 (3) Å to H18A. The differences in the O^···^H contacts are reflected by the different Fe—O—Fe angles.

The packing of the title compound consists of layers of cations, the anions lay between the layers as shown in Fig. 2. Several H^···^Cl hydrogen bonds are connecting the cations and anions. The shortest H^···^Cl contact is 2.686Å.

### Experimental

Addition of a warm solution of (C_14_H_34_N_2_)Cl_2_ (0.028 g, 0.093 mmol) in 2 ml EtOH to a solution of FeCl_2_^.^4H_2_O (0.021 g, 0.106 mmol) in 3 ml EtOH gave a clear solution from which a brown solid precipitated. After filtering the solution, yellow plates of the title compound were grown from the filtrate.

### Refinement

H atoms were positioned geometrically and refined using a riding model with C—H = 0.99 Å and with *U*_iso_(H) = 1.2 *U*_eq_(C) and 0.98 Å and *U*_iso_(H) = 1.5 *U*_eq_(C) for the methylene and methyl H atoms, respectively.

### Crystal data

**Table:**

(C_14_H_34_N_2_)[Fe_2_Cl_6_O]	*Z* = 4
*M**_r_* = 570.83	*F*(000) = 1176
Triclinic, *P*1	*D*_x_ = 1.531 Mg m^−^^3^
Hall symbol: -P 1	Mo *K*α radiation, λ = 0.71073 Å
*a* = 8.9803 (18) Å	Cell parameters from 7321 reflections
*b* = 14.689 (3) Å	θ = 2.3–25.0°
*c* = 19.249 (4) Å	µ = 1.83 mm^−^^1^
α = 81.75 (3)°	*T* = 120 K
β = 87.66 (3)°	Plate, yellow
γ = 80.32 (3)°	0.25 × 0.10 × 0.08 mm
*V* = 2476.8 (9) Å^3^	

### Data collection

**Table:**

Bruker–Nonius KappaCCD diffractometer	8498 independent reflections
Radiation source: fine-focus sealed tube	7321 reflections with *I* > 2σ(*I*)
Graphite monochromator	*R*_int_ = 0.096
φ scans, and ω scans with κ offsets	θ_max_ = 25.0°, θ_min_ = 2.3°
Absorption correction: multi-scan (*SHELXTL*; Sheldrick, 2008)	*h* = −10→10
*T*_min_ = 0.658, *T*_max_ = 0.868	*k* = −17→17
29910 measured reflections	*l* = −22→22

### Refinement

**Table:**

Refinement on *F*^2^	Secondary atom site location: difference Fourier map
Least-squares matrix: full	Hydrogen site location: inferred from neighbouring sites
*R*[*F*^2^ > 2σ(*F*^2^)] = 0.045	H-atom parameters constrained
*wR*(*F*^2^) = 0.128	*w* = 1/[σ^2^(*F*_o_^2^) + (0.0711*P*)^2^ + 1.9303*P*] where *P* = (*F*_o_^2^ + 2*F*_c_^2^)/3
*S* = 1.12	(Δ/σ)_max_ = 0.001
8498 reflections	Δρ_max_ = 0.85 e Å^−^^3^
464 parameters	Δρ_min_ = −0.81 e Å^−^^3^
0 restraints	Extinction correction: *SHELXL97* (Sheldrick, 2008), Fc^*^=kFc[1+0.001xFc^2^λ^3^/sin(2θ)]^-1/4^
Primary atom site location: structure-invariant direct methods	Extinction coefficient: 0.0242 (10)

### Special details

**Table:**

Geometry. All s.u.'s (except the s.u. in the dihedral angle between two l.s. planes) are estimated using the full covariance matrix. The cell s.u.'s are taken into account individually in the estimation of s.u.'s in distances, angles and torsion angles; correlations between s.u.'s in cell parameters are only used when they are defined by crystal symmetry. An approximate (isotropic) treatment of cell s.u.'s is used for estimating s.u.'s involving l.s. planes.
Refinement. Refinement of *F*^2^ against ALL reflections. The weighted *R*-factor *wR* and goodness of fit *S* are based on *F*^2^, conventional *R*-factors *R* are based on *F*, with *F* set to zero for negative *F*^2^. The threshold expression of *F*^2^ > 2σ(*F*^2^) is used only for calculating *R*-factors(gt) *etc*. and is not relevant to the choice of reflections for refinement. *R*-factors based on *F*^2^ are statistically about twice as large as those based on *F*, and *R*- factors based on ALL data will be even larger.

### Fractional atomic coordinates and isotropic or equivalent isotropic displacement parameters (Å^2^)

**Table:**

	*x*	*y*	*z*	*U*_iso_*/*U*_eq_	
Fe1	0.10874 (5)	0.75030 (3)	0.95062 (3)	0.01283 (15)	
Fe2	0.37263 (5)	0.74978 (3)	0.82154 (3)	0.01384 (15)	
Fe3	0.09056 (6)	0.28369 (4)	0.56365 (3)	0.01490 (15)	
Fe4	0.36880 (6)	0.23368 (3)	0.68992 (3)	0.01514 (15)	
Cl11	0.07970 (10)	0.63429 (6)	1.03616 (4)	0.0176 (2)	
Cl12	0.13988 (11)	0.87485 (6)	0.99662 (5)	0.0240 (2)	
Cl13	−0.09395 (10)	0.78206 (6)	0.88423 (5)	0.0184 (2)	
Cl21	0.50743 (10)	0.85314 (6)	0.84802 (5)	0.0218 (2)	
Cl22	0.22807 (10)	0.81162 (7)	0.72944 (5)	0.0233 (2)	
Cl23	0.52966 (10)	0.62497 (6)	0.79333 (5)	0.0215 (2)	
Cl31	0.00871 (14)	0.14964 (7)	0.55993 (5)	0.0325 (3)	
Cl32	0.21043 (12)	0.32056 (7)	0.46229 (5)	0.0263 (2)	
Cl33	−0.10178 (11)	0.39595 (8)	0.57735 (5)	0.0305 (3)	
Cl41	0.58302 (11)	0.22816 (7)	0.62632 (5)	0.0262 (2)	
Cl42	0.35503 (11)	0.09363 (6)	0.74772 (5)	0.0234 (2)	
Cl43	0.37577 (11)	0.33105 (7)	0.76780 (5)	0.0250 (2)	
O1	0.2675 (3)	0.71338 (18)	0.89740 (13)	0.0177 (5)	
O2	0.2137 (3)	0.2739 (2)	0.63388 (14)	0.0261 (6)	
N1	0.8834 (3)	0.0750 (2)	0.78431 (16)	0.0153 (6)	
N2	0.7825 (3)	−0.1068 (2)	0.67535 (15)	0.0145 (6)	
N3	0.6006 (3)	0.4534 (2)	0.41794 (16)	0.0155 (6)	
N4	0.6092 (3)	0.5954 (2)	0.02212 (15)	0.0124 (6)	
C1	0.8655 (4)	0.0313 (3)	0.71950 (19)	0.0184 (8)	
H1A	0.9580	0.0325	0.6898	0.022*	
H1B	0.7798	0.0687	0.6921	0.022*	
C2	0.8375 (4)	−0.0689 (2)	0.73682 (18)	0.0149 (7)	
H2A	0.9326	−0.1090	0.7537	0.018*	
H2B	0.7618	−0.0723	0.7755	0.018*	
C3	1.0087 (4)	0.0191 (3)	0.8295 (2)	0.0222 (8)	
H3A	1.0201	0.0507	0.8700	0.033*	
H3B	1.1033	0.0132	0.8021	0.033*	
H3C	0.9843	−0.0431	0.8459	0.033*	
C4	0.7392 (4)	0.0853 (3)	0.8260 (2)	0.0261 (9)	
H4A	0.6561	0.1163	0.7952	0.039*	
H4B	0.7478	0.1228	0.8634	0.039*	
H4C	0.7190	0.0236	0.8467	0.039*	
C5	0.9219 (4)	0.1720 (2)	0.7566 (2)	0.0193 (8)	
H5A	1.0082	0.1643	0.7231	0.023*	
H5B	0.8344	0.2089	0.7303	0.023*	
C6	0.9615 (5)	0.2272 (3)	0.8122 (2)	0.0222 (8)	
H6A	1.0660	0.2032	0.8282	0.027*	
H6B	0.8921	0.2204	0.8532	0.027*	
C7	0.9478 (4)	0.3304 (2)	0.7807 (2)	0.0185 (8)	
H7A	1.0028	0.3629	0.8104	0.022*	
H7B	0.9958	0.3350	0.7334	0.022*	
C8	0.7839 (5)	0.3788 (3)	0.7751 (2)	0.0279 (9)	
H8A	0.7289	0.3469	0.7457	0.042*	
H8B	0.7804	0.4439	0.7539	0.042*	
H8C	0.7370	0.3767	0.8220	0.042*	
C9	0.7838 (4)	−0.2093 (2)	0.69915 (19)	0.0175 (8)	
H9A	0.7515	−0.2380	0.6607	0.026*	
H9B	0.7145	−0.2171	0.7394	0.026*	
H9C	0.8863	−0.2393	0.7128	0.026*	
C10	0.8884 (4)	−0.0971 (3)	0.61358 (19)	0.0191 (8)	
H10A	0.8793	−0.0313	0.5934	0.029*	
H10B	0.8629	−0.1334	0.5781	0.029*	
H10C	0.9924	−0.1203	0.6288	0.029*	
C11	0.6223 (4)	−0.0583 (3)	0.6572 (2)	0.0174 (8)	
H11A	0.5613	−0.0578	0.7012	0.021*	
H11B	0.6261	0.0074	0.6377	0.021*	
C12	0.5419 (4)	−0.1008 (3)	0.6057 (2)	0.0200 (8)	
H12A	0.5230	−0.1634	0.6271	0.024*	
H12B	0.6059	−0.1080	0.5631	0.024*	
C13	0.3919 (4)	−0.0378 (3)	0.5859 (2)	0.0208 (8)	
H13A	0.3337	−0.0252	0.6293	0.025*	
H13B	0.4124	0.0225	0.5606	0.025*	
C14	0.2976 (5)	−0.0812 (3)	0.5399 (2)	0.0243 (9)	
H14A	0.3556	−0.0950	0.4974	0.036*	
H14B	0.2047	−0.0376	0.5270	0.036*	
H14C	0.2718	−0.1391	0.5659	0.036*	
C15	0.5689 (4)	0.5107 (2)	0.47771 (19)	0.0163 (7)	
H15A	0.5525	0.5777	0.4583	0.020*	
H15B	0.6585	0.4985	0.5081	0.020*	
C16	0.6160 (5)	0.3507 (2)	0.4452 (2)	0.0209 (8)	
H16A	0.5171	0.3358	0.4618	0.031*	
H16B	0.6870	0.3353	0.4841	0.031*	
H16C	0.6538	0.3143	0.4074	0.031*	
C17	0.4756 (4)	0.4807 (3)	0.3655 (2)	0.0221 (8)	
H17A	0.4950	0.4414	0.3279	0.033*	
H17B	0.4715	0.5462	0.3454	0.033*	
H17C	0.3790	0.4722	0.3889	0.033*	
C18	0.7507 (4)	0.4745 (2)	0.38445 (19)	0.0162 (7)	
H18A	0.7461	0.5430	0.3758	0.019*	
H18B	0.8322	0.4489	0.4184	0.019*	
C19	0.7923 (4)	0.4357 (3)	0.3159 (2)	0.0192 (8)	
H19A	0.7243	0.4705	0.2784	0.023*	
H19B	0.7796	0.3694	0.3214	0.023*	
C20	0.9559 (4)	0.4442 (3)	0.2953 (2)	0.0204 (8)	
H20A	0.9733	0.5080	0.2993	0.025*	
H20B	1.0244	0.3998	0.3280	0.025*	
C21	0.9924 (4)	0.4241 (3)	0.2206 (2)	0.0228 (8)	
H21A	0.9630	0.3644	0.2148	0.034*	
H21B	1.1012	0.4211	0.2114	0.034*	
H21C	0.9367	0.4740	0.1876	0.034*	
C22	0.4764 (4)	0.5500 (2)	0.0076 (2)	0.0156 (7)	
H22A	0.4210	0.5875	−0.0331	0.019*	
H22B	0.4066	0.5502	0.0488	0.019*	
C23	0.6971 (4)	0.5408 (3)	0.08372 (19)	0.0181 (8)	
H23A	0.7482	0.4809	0.0710	0.027*	
H23B	0.6280	0.5296	0.1236	0.027*	
H23C	0.7725	0.5762	0.0967	0.027*	
C24	0.7133 (4)	0.6047 (2)	−0.04055 (18)	0.0156 (7)	
H24A	0.8004	0.6312	−0.0285	0.023*	
H24B	0.6593	0.6459	−0.0793	0.023*	
H24C	0.7482	0.5431	−0.0548	0.023*	
C25	0.5408 (4)	0.6924 (2)	0.0395 (2)	0.0151 (7)	
H25A	0.4625	0.7208	0.0046	0.018*	
H25B	0.4899	0.6851	0.0861	0.018*	
C26	0.6520 (4)	0.7591 (2)	0.0406 (2)	0.0165 (7)	
H26A	0.6981	0.7713	−0.0067	0.020*	
H26B	0.7337	0.7308	0.0738	0.020*	
C27	0.5707 (4)	0.8507 (2)	0.0629 (2)	0.0188 (8)	
H27A	0.4772	0.8722	0.0356	0.023*	
H27B	0.5418	0.8402	0.1132	0.023*	
C28	0.6706 (5)	0.9256 (3)	0.0511 (2)	0.0252 (9)	
H28A	0.7666	0.9025	0.0750	0.038*	
H28B	0.6196	0.9815	0.0700	0.038*	
H28C	0.6899	0.9411	0.0007	0.038*	

### Atomic displacement parameters (Å^2^)

**Table:**

	*U*^11^	*U*^22^	*U*^33^	*U*^12^	*U*^13^	*U*^23^
Fe1	0.0126 (3)	0.0130 (3)	0.0124 (3)	−0.0023 (2)	−0.00025 (19)	−0.00008 (19)
Fe2	0.0127 (3)	0.0154 (3)	0.0127 (3)	−0.0016 (2)	0.0003 (2)	−0.0004 (2)
Fe3	0.0150 (3)	0.0164 (3)	0.0131 (3)	−0.0016 (2)	−0.0023 (2)	−0.0022 (2)
Fe4	0.0144 (3)	0.0163 (3)	0.0140 (3)	−0.0004 (2)	−0.0028 (2)	−0.0017 (2)
Cl11	0.0180 (4)	0.0168 (4)	0.0158 (4)	−0.0018 (3)	0.0012 (3)	0.0031 (3)
Cl12	0.0327 (5)	0.0193 (5)	0.0226 (5)	−0.0097 (4)	−0.0018 (4)	−0.0052 (4)
Cl13	0.0160 (4)	0.0217 (5)	0.0165 (4)	−0.0051 (4)	−0.0029 (3)	0.0035 (3)
Cl21	0.0215 (5)	0.0217 (5)	0.0241 (5)	−0.0072 (4)	0.0016 (4)	−0.0061 (4)
Cl22	0.0212 (5)	0.0275 (5)	0.0185 (5)	−0.0010 (4)	−0.0054 (4)	0.0037 (4)
Cl23	0.0203 (5)	0.0228 (5)	0.0214 (5)	0.0015 (4)	−0.0013 (4)	−0.0085 (4)
Cl31	0.0525 (7)	0.0274 (5)	0.0234 (5)	−0.0223 (5)	0.0107 (5)	−0.0081 (4)
Cl32	0.0323 (5)	0.0296 (5)	0.0205 (5)	−0.0148 (4)	0.0066 (4)	−0.0063 (4)
Cl33	0.0237 (5)	0.0360 (6)	0.0277 (5)	0.0124 (4)	−0.0075 (4)	−0.0092 (4)
Cl41	0.0198 (5)	0.0324 (5)	0.0235 (5)	−0.0017 (4)	0.0040 (4)	0.0024 (4)
Cl42	0.0256 (5)	0.0182 (5)	0.0261 (5)	−0.0043 (4)	0.0040 (4)	−0.0018 (4)
Cl43	0.0345 (5)	0.0234 (5)	0.0195 (5)	−0.0073 (4)	−0.0038 (4)	−0.0065 (4)
O1	0.0159 (12)	0.0206 (13)	0.0156 (13)	−0.0024 (11)	0.0021 (10)	−0.0003 (10)
O2	0.0223 (14)	0.0336 (16)	0.0210 (15)	0.0040 (12)	−0.0064 (12)	−0.0073 (12)
N1	0.0129 (15)	0.0187 (16)	0.0145 (15)	−0.0030 (12)	−0.0024 (12)	−0.0019 (12)
N2	0.0155 (15)	0.0138 (15)	0.0140 (15)	−0.0018 (12)	0.0007 (12)	−0.0022 (12)
N3	0.0174 (15)	0.0152 (15)	0.0144 (15)	−0.0022 (12)	−0.0016 (12)	−0.0044 (12)
N4	0.0135 (14)	0.0113 (14)	0.0136 (15)	−0.0036 (12)	−0.0012 (12)	−0.0036 (11)
C1	0.0233 (19)	0.0197 (19)	0.0140 (18)	−0.0053 (16)	−0.0023 (15)	−0.0055 (14)
C2	0.0173 (18)	0.0162 (18)	0.0112 (17)	−0.0021 (14)	−0.0023 (14)	−0.0019 (14)
C3	0.025 (2)	0.0156 (19)	0.025 (2)	−0.0057 (16)	−0.0121 (17)	0.0044 (15)
C4	0.021 (2)	0.038 (2)	0.025 (2)	−0.0123 (18)	0.0092 (17)	−0.0160 (18)
C5	0.025 (2)	0.0128 (18)	0.0194 (19)	−0.0013 (15)	−0.0032 (16)	−0.0010 (14)
C6	0.031 (2)	0.0187 (19)	0.0174 (19)	−0.0064 (17)	−0.0053 (16)	−0.0004 (15)
C7	0.023 (2)	0.0151 (18)	0.0187 (19)	−0.0057 (15)	0.0000 (15)	−0.0041 (15)
C8	0.022 (2)	0.026 (2)	0.036 (2)	−0.0033 (17)	−0.0035 (18)	−0.0077 (18)
C9	0.0213 (19)	0.0133 (18)	0.0164 (18)	−0.0014 (15)	−0.0017 (15)	0.0017 (14)
C10	0.0201 (19)	0.024 (2)	0.0127 (18)	−0.0037 (16)	0.0031 (15)	−0.0037 (15)
C11	0.0164 (18)	0.0153 (18)	0.0192 (19)	0.0007 (15)	−0.0037 (15)	−0.0011 (14)
C12	0.0200 (19)	0.0200 (19)	0.0201 (19)	−0.0017 (16)	−0.0025 (15)	−0.0041 (15)
C13	0.022 (2)	0.021 (2)	0.0184 (19)	−0.0044 (16)	−0.0028 (16)	0.0000 (15)
C14	0.025 (2)	0.027 (2)	0.022 (2)	−0.0066 (17)	−0.0052 (16)	0.0003 (16)
C15	0.0194 (18)	0.0162 (18)	0.0145 (18)	−0.0039 (15)	0.0028 (15)	−0.0062 (14)
C16	0.028 (2)	0.0114 (18)	0.023 (2)	−0.0042 (16)	0.0067 (16)	−0.0040 (15)
C17	0.0190 (19)	0.031 (2)	0.0173 (19)	−0.0036 (16)	−0.0055 (15)	−0.0062 (16)
C18	0.0154 (17)	0.0160 (18)	0.0170 (18)	−0.0030 (14)	0.0008 (14)	−0.0016 (14)
C19	0.0195 (19)	0.0199 (19)	0.0190 (19)	−0.0032 (15)	0.0006 (15)	−0.0055 (15)
C20	0.022 (2)	0.0184 (19)	0.020 (2)	−0.0013 (16)	0.0011 (16)	−0.0040 (15)
C21	0.024 (2)	0.021 (2)	0.022 (2)	−0.0009 (16)	0.0014 (16)	−0.0043 (16)
C22	0.0144 (17)	0.0114 (18)	0.0222 (19)	−0.0053 (14)	−0.0047 (15)	−0.0012 (14)
C23	0.0216 (19)	0.0157 (18)	0.0162 (18)	−0.0021 (15)	−0.0067 (15)	0.0009 (14)
C24	0.0185 (18)	0.0165 (18)	0.0128 (17)	−0.0050 (15)	0.0045 (14)	−0.0036 (14)
C25	0.0142 (17)	0.0088 (17)	0.0224 (19)	−0.0001 (14)	0.0011 (14)	−0.0051 (14)
C26	0.0163 (18)	0.0139 (18)	0.0203 (19)	−0.0035 (15)	−0.0027 (15)	−0.0042 (14)
C27	0.0200 (19)	0.0152 (18)	0.022 (2)	−0.0053 (15)	0.0016 (15)	−0.0046 (15)
C28	0.029 (2)	0.0175 (19)	0.032 (2)	−0.0095 (17)	−0.0013 (18)	−0.0070 (17)

### Geometric parameters (Å, º)

**Table:**

Fe1—O1	1.776 (3)	C10—H10A	0.9800
Fe1—Cl12	2.2060 (12)	C10—H10B	0.9800
Fe1—Cl13	2.2130 (12)	C10—H10C	0.9800
Fe1—Cl11	2.2344 (12)	C11—C12	1.511 (5)
Fe2—O1	1.773 (3)	C11—H11A	0.9900
Fe2—Cl21	2.2183 (12)	C11—H11B	0.9900
Fe2—Cl22	2.2321 (13)	C12—C13	1.529 (5)
Fe2—Cl23	2.2356 (13)	C12—H12A	0.9900
Fe3—O2	1.756 (3)	C12—H12B	0.9900
Fe3—Cl33	2.2141 (13)	C13—C14	1.520 (5)
Fe3—Cl31	2.2257 (12)	C13—H13A	0.9900
Fe3—Cl32	2.2316 (13)	C13—H13B	0.9900
Fe4—O2	1.765 (3)	C14—H14A	0.9800
Fe4—Cl42	2.2142 (12)	C14—H14B	0.9800
Fe4—Cl43	2.2248 (12)	C14—H14C	0.9800
Fe4—Cl41	2.2338 (13)	C15—C15^i^	1.528 (7)
N1—C4	1.492 (5)	C15—H15A	0.9900
N1—C3	1.504 (4)	C15—H15B	0.9900
N1—C1	1.508 (4)	C16—H16A	0.9800
N1—C5	1.538 (5)	C16—H16B	0.9800
N2—C10	1.498 (5)	C16—H16C	0.9800
N2—C9	1.508 (5)	C17—H17A	0.9800
N2—C2	1.511 (4)	C17—H17B	0.9800
N2—C11	1.525 (4)	C17—H17C	0.9800
N3—C17	1.505 (5)	C18—C19	1.520 (5)
N3—C16	1.509 (5)	C18—H18A	0.9900
N3—C15	1.511 (4)	C18—H18B	0.9900
N3—C18	1.531 (5)	C19—C20	1.527 (5)
N4—C24	1.500 (5)	C19—H19A	0.9900
N4—C23	1.505 (4)	C19—H19B	0.9900
N4—C22	1.514 (4)	C20—C21	1.520 (5)
N4—C25	1.531 (4)	C20—H20A	0.9900
C1—C2	1.522 (5)	C20—H20B	0.9900
C1—H1A	0.9900	C21—H21A	0.9800
C1—H1B	0.9900	C21—H21B	0.9800
C2—H2A	0.9900	C21—H21C	0.9800
C2—H2B	0.9900	C22—C22^ii^	1.527 (7)
C3—H3A	0.9800	C22—H22A	0.9900
C3—H3B	0.9800	C22—H22B	0.9900
C3—H3C	0.9800	C23—H23A	0.9800
C4—H4A	0.9800	C23—H23B	0.9800
C4—H4B	0.9800	C23—H23C	0.9800
C4—H4C	0.9800	C24—H24A	0.9800
C5—C6	1.518 (5)	C24—H24B	0.9800
C5—H5A	0.9900	C24—H24C	0.9800
C5—H5B	0.9900	C25—C26	1.514 (5)
C6—C7	1.537 (5)	C25—H25A	0.9900
C6—H6A	0.9900	C25—H25B	0.9900
C6—H6B	0.9900	C26—C27	1.529 (5)
C7—C8	1.524 (5)	C26—H26A	0.9900
C7—H7A	0.9900	C26—H26B	0.9900
C7—H7B	0.9900	C27—C28	1.519 (5)
C8—H8A	0.9800	C27—H27A	0.9900
C8—H8B	0.9800	C27—H27B	0.9900
C8—H8C	0.9800	C28—H28A	0.9800
C9—H9A	0.9800	C28—H28B	0.9800
C9—H9B	0.9800	C28—H28C	0.9800
C9—H9C	0.9800		
			
O1—Fe1—Cl12	110.70 (9)	H10B—C10—H10C	109.5
O1—Fe1—Cl13	108.20 (9)	C12—C11—N2	115.8 (3)
Cl12—Fe1—Cl13	110.14 (5)	C12—C11—H11A	108.3
O1—Fe1—Cl11	108.96 (9)	N2—C11—H11A	108.3
Cl12—Fe1—Cl11	109.78 (4)	C12—C11—H11B	108.3
Cl13—Fe1—Cl11	109.00 (5)	N2—C11—H11B	108.3
O1—Fe2—Cl21	108.21 (9)	H11A—C11—H11B	107.4
O1—Fe2—Cl22	113.20 (9)	C11—C12—C13	109.4 (3)
Cl21—Fe2—Cl22	109.79 (5)	C11—C12—H12A	109.8
O1—Fe2—Cl23	107.89 (9)	C13—C12—H12A	109.8
Cl21—Fe2—Cl23	109.02 (4)	C11—C12—H12B	109.8
Cl22—Fe2—Cl23	108.65 (5)	C13—C12—H12B	109.8
O2—Fe3—Cl33	108.14 (10)	H12A—C12—H12B	108.2
O2—Fe3—Cl31	110.99 (11)	C14—C13—C12	112.2 (3)
Cl33—Fe3—Cl31	110.46 (5)	C14—C13—H13A	109.2
O2—Fe3—Cl32	109.89 (10)	C12—C13—H13A	109.2
Cl33—Fe3—Cl32	109.28 (5)	C14—C13—H13B	109.2
Cl31—Fe3—Cl32	108.07 (5)	C12—C13—H13B	109.2
O2—Fe4—Cl42	111.31 (11)	H13A—C13—H13B	107.9
O2—Fe4—Cl43	110.40 (10)	C13—C14—H14A	109.5
Cl42—Fe4—Cl43	108.37 (4)	C13—C14—H14B	109.5
O2—Fe4—Cl41	109.30 (10)	H14A—C14—H14B	109.5
Cl42—Fe4—Cl41	109.55 (5)	C13—C14—H14C	109.5
Cl43—Fe4—Cl41	107.84 (5)	H14A—C14—H14C	109.5
Fe2—O1—Fe1	142.68 (16)	H14B—C14—H14C	109.5
Fe3—O2—Fe4	160.84 (18)	N3—C15—C15^i^	112.9 (4)
C4—N1—C3	109.8 (3)	N3—C15—H15A	109.0
C4—N1—C1	110.7 (3)	C15^i^—C15—H15A	109.0
C3—N1—C1	111.6 (3)	N3—C15—H15B	109.0
C4—N1—C5	109.6 (3)	C15^i^—C15—H15B	109.0
C3—N1—C5	110.1 (3)	H15A—C15—H15B	107.8
C1—N1—C5	105.0 (3)	N3—C16—H16A	109.5
C10—N2—C9	108.2 (3)	N3—C16—H16B	109.5
C10—N2—C2	110.9 (3)	H16A—C16—H16B	109.5
C9—N2—C2	105.9 (3)	N3—C16—H16C	109.5
C10—N2—C11	111.7 (3)	H16A—C16—H16C	109.5
C9—N2—C11	110.1 (3)	H16B—C16—H16C	109.5
C2—N2—C11	109.9 (3)	N3—C17—H17A	109.5
C17—N3—C16	110.0 (3)	N3—C17—H17B	109.5
C17—N3—C15	110.0 (3)	H17A—C17—H17B	109.5
C16—N3—C15	110.5 (3)	N3—C17—H17C	109.5
C17—N3—C18	110.7 (3)	H17A—C17—H17C	109.5
C16—N3—C18	109.4 (3)	H17B—C17—H17C	109.5
C15—N3—C18	106.1 (3)	C19—C18—N3	114.9 (3)
C24—N4—C23	109.2 (3)	C19—C18—H18A	108.5
C24—N4—C22	111.8 (3)	N3—C18—H18A	108.5
C23—N4—C22	110.9 (3)	C19—C18—H18B	108.5
C24—N4—C25	109.6 (3)	N3—C18—H18B	108.5
C23—N4—C25	109.7 (3)	H18A—C18—H18B	107.5
C22—N4—C25	105.7 (3)	C18—C19—C20	110.0 (3)
N1—C1—C2	112.5 (3)	C18—C19—H19A	109.7
N1—C1—H1A	109.1	C20—C19—H19A	109.7
C2—C1—H1A	109.1	C18—C19—H19B	109.7
N1—C1—H1B	109.1	C20—C19—H19B	109.7
C2—C1—H1B	109.1	H19A—C19—H19B	108.2
H1A—C1—H1B	107.8	C21—C20—C19	111.5 (3)
N2—C2—C1	113.6 (3)	C21—C20—H20A	109.3
N2—C2—H2A	108.8	C19—C20—H20A	109.3
C1—C2—H2A	108.8	C21—C20—H20B	109.3
N2—C2—H2B	108.8	C19—C20—H20B	109.3
C1—C2—H2B	108.8	H20A—C20—H20B	108.0
H2A—C2—H2B	107.7	C20—C21—H21A	109.5
N1—C3—H3A	109.5	C20—C21—H21B	109.5
N1—C3—H3B	109.5	H21A—C21—H21B	109.5
H3A—C3—H3B	109.5	C20—C21—H21C	109.5
N1—C3—H3C	109.5	H21A—C21—H21C	109.5
H3A—C3—H3C	109.5	H21B—C21—H21C	109.5
H3B—C3—H3C	109.5	N4—C22—C22^ii^	112.9 (4)
N1—C4—H4A	109.5	N4—C22—H22A	109.0
N1—C4—H4B	109.5	C22^ii^—C22—H22A	109.0
H4A—C4—H4B	109.5	N4—C22—H22B	109.0
N1—C4—H4C	109.5	C22^ii^—C22—H22B	109.0
H4A—C4—H4C	109.5	H22A—C22—H22B	107.8
H4B—C4—H4C	109.5	N4—C23—H23A	109.5
C6—C5—N1	115.5 (3)	N4—C23—H23B	109.5
C6—C5—H5A	108.4	H23A—C23—H23B	109.5
N1—C5—H5A	108.4	N4—C23—H23C	109.5
C6—C5—H5B	108.4	H23A—C23—H23C	109.5
N1—C5—H5B	108.4	H23B—C23—H23C	109.5
H5A—C5—H5B	107.5	N4—C24—H24A	109.5
C5—C6—C7	109.0 (3)	N4—C24—H24B	109.5
C5—C6—H6A	109.9	H24A—C24—H24B	109.5
C7—C6—H6A	109.9	N4—C24—H24C	109.5
C5—C6—H6B	109.9	H24A—C24—H24C	109.5
C7—C6—H6B	109.9	H24B—C24—H24C	109.5
H6A—C6—H6B	108.3	C26—C25—N4	115.3 (3)
C8—C7—C6	112.3 (3)	C26—C25—H25A	108.4
C8—C7—H7A	109.2	N4—C25—H25A	108.4
C6—C7—H7A	109.2	C26—C25—H25B	108.4
C8—C7—H7B	109.2	N4—C25—H25B	108.4
C6—C7—H7B	109.2	H25A—C25—H25B	107.5
H7A—C7—H7B	107.9	C25—C26—C27	109.9 (3)
C7—C8—H8A	109.5	C25—C26—H26A	109.7
C7—C8—H8B	109.5	C27—C26—H26A	109.7
H8A—C8—H8B	109.5	C25—C26—H26B	109.7
C7—C8—H8C	109.5	C27—C26—H26B	109.7
H8A—C8—H8C	109.5	H26A—C26—H26B	108.2
H8B—C8—H8C	109.5	C28—C27—C26	111.3 (3)
N2—C9—H9A	109.5	C28—C27—H27A	109.4
N2—C9—H9B	109.5	C26—C27—H27A	109.4
H9A—C9—H9B	109.5	C28—C27—H27B	109.4
N2—C9—H9C	109.5	C26—C27—H27B	109.4
H9A—C9—H9C	109.5	H27A—C27—H27B	108.0
H9B—C9—H9C	109.5	C27—C28—H28A	109.5
N2—C10—H10A	109.5	C27—C28—H28B	109.5
N2—C10—H10B	109.5	H28A—C28—H28B	109.5
H10A—C10—H10B	109.5	C27—C28—H28C	109.5
N2—C10—H10C	109.5	H28A—C28—H28C	109.5
H10A—C10—H10C	109.5	H28B—C28—H28C	109.5
			
Cl21—Fe2—O1—Fe1	73.9 (3)	C5—C6—C7—C8	76.4 (4)
Cl22—Fe2—O1—Fe1	−48.0 (3)	C10—N2—C11—C12	67.2 (4)
Cl23—Fe2—O1—Fe1	−168.3 (2)	C9—N2—C11—C12	−53.0 (4)
Cl12—Fe1—O1—Fe2	−61.2 (3)	C2—N2—C11—C12	−169.2 (3)
Cl13—Fe1—O1—Fe2	59.5 (3)	N2—C11—C12—C13	−172.8 (3)
Cl11—Fe1—O1—Fe2	177.9 (2)	C11—C12—C13—C14	−174.2 (3)
Cl33—Fe3—O2—Fe4	177.5 (6)	C17—N3—C15—C15^i^	66.8 (5)
Cl31—Fe3—O2—Fe4	56.2 (6)	C16—N3—C15—C15^i^	−54.9 (5)
Cl32—Fe3—O2—Fe4	−63.3 (6)	C18—N3—C15—C15^i^	−173.4 (4)
Cl42—Fe4—O2—Fe3	−68.8 (6)	C17—N3—C18—C19	−51.5 (4)
Cl43—Fe4—O2—Fe3	170.8 (5)	C16—N3—C18—C19	69.9 (4)
Cl41—Fe4—O2—Fe3	52.4 (6)	C15—N3—C18—C19	−170.9 (3)
C4—N1—C1—C2	−65.9 (4)	N3—C18—C19—C20	−168.9 (3)
C3—N1—C1—C2	56.7 (4)	C18—C19—C20—C21	−168.8 (3)
C5—N1—C1—C2	176.0 (3)	C24—N4—C22—C22^ii^	63.4 (5)
C10—N2—C2—C1	54.7 (4)	C23—N4—C22—C22^ii^	−58.7 (5)
C9—N2—C2—C1	171.8 (3)	C25—N4—C22—C22^ii^	−177.4 (4)
C11—N2—C2—C1	−69.4 (4)	C24—N4—C25—C26	−46.7 (4)
N1—C1—C2—N2	166.2 (3)	C23—N4—C25—C26	73.1 (4)
C4—N1—C5—C6	67.1 (4)	C22—N4—C25—C26	−167.3 (3)
C3—N1—C5—C6	−53.8 (4)	N4—C25—C26—C27	−176.6 (3)
C1—N1—C5—C6	−174.0 (3)	C25—C26—C27—C28	−169.0 (3)
N1—C5—C6—C7	−162.3 (3)		

Symmetry codes: (i) −*x*+1, −*y*+1, −*z*+1; (ii) −*x*+1, −*y*+1, −*z*.

### Hydrogen-bond geometry (Å, º)

**Table:**

*D*—H···*A*	*D*—H	H···*A*	*D*···*A*	*D*—H···*A*
C2—H2*A*···Cl13^iii^	0.99	2.80	3.336 (3)	114
C2—H2*A*···Cl22^iii^	0.99	2.76	3.656 (4)	150
C3—H3*B*···Cl42^iv^	0.98	2.82	3.688 (4)	148
C3—H3*C*···Cl13^iii^	0.98	2.77	3.746 (4)	176
C9—H9*A*···Cl32^v^	0.98	2.79	3.708 (4)	155
C15—H15*B*···Cl33^iv^	0.99	2.69	3.623 (4)	157
C16—H16*A*···Cl32	0.98	2.80	3.738 (5)	160
C17—H17*A*···Cl23^i^	0.98	2.69	3.627 (4)	161
C22—H22*A*···O1^vi^	0.99	2.39	3.336 (4)	161
C24—H24*C*···Cl11^i^	0.98	2.78	3.677 (3)	152
C26—H26*A*···Cl13^vii^	0.99	2.76	3.713 (4)	162

Symmetry codes: (i) −*x*+1, −*y*+1, −*z*+1; (iii) *x*+1, *y*−1, *z*; (iv) *x*+1, *y*, *z*; (v) −*x*+1, −*y*, −*z*+1; (vi) *x*, *y*, *z*−1; (vii) *x*+1, *y*, *z*−1.
